# Beyond Clinical Skills: What Shapes Job Performance Among ICU Respiratory Therapists?

**DOI:** 10.3390/healthcare14081007

**Published:** 2026-04-11

**Authors:** Rayan A. Siraj, Maryam M. Almulhem, Ibrahim A. Elshaer

**Affiliations:** 1 Department of Respiratory Therapy, College of Applied Medical Sciences, King Faisal University, Al-Ahsa 31982, Saudi Arabia; mmalmulhim@kfu.edu.sa; 2 Department of Management, College of Business Administration, King Faisal University, Al-Ahsa 31982, Saudi Arabia; ielshaer@kfu.edu.sa

**Keywords:** emotional intelligence, intensive care units, job performance, respiratory therapists, work–life balance

## Abstract

Background: Intensive care units (ICUs) are high-acuity environments that require respiratory therapists (RTs) to maintain vigilance, manage emotions, and make rapid clinical decisions. In such settings, performance stability is critical for patient safety. Although emotional intelligence (EI) and work–life balance (WLB) have been linked to professional outcomes in health care, their independent and direction-specific associations with job performance among ICU respiratory therapists remain underexamined. Methods: A national cross-sectional survey was conducted among respiratory therapists working in ICUs across Saudi Arabia (June 2025–January 2026). EI was measured using the Wong and Law Emotional Intelligence Scale. WLB was assessed using the work interference with personal life (WIPL), personal life interference with work (PLIW), and work–personal life enhancement (WPLE) scales. Job performance was evaluated using the Individual Work Performance Questionnaire. Correlation and multivariable linear regression analyses were performed to estimate independent associations. Results: A total of 392 RTs were included in the final analysis. Higher EI was independently associated with greater task performance (B = 0.21, *p* < 0.01) and contextual performance (B = 0.30, *p* < 0.001), and with lower counterproductive work behaviours (B = −0.24, *p* < 0.001). Among WLB dimensions, PLIW showed the strongest adverse association, predicting lower task performance (B = −0.20, *p* < 0.05) and higher counterproductive behaviours (B = 0.39, *p* < 0.001), but was not significantly associated with contextual performance in the fully adjusted model. WPLE demonstrated modest positive associations with performance, whereas WIPL was not significant in adjusted models. Conclusions: Job performance among ICU respiratory therapists is shaped by both emotional regulatory capacity and cross-domain strain. Personal life interference with work emerged as the most influential adverse predictor, whereas EI was associated with constructive performance patterns. Findings should be interpreted in light of the cross-sectional design and self-reported data. Sustaining performance in high-acuity settings requires attention to emotional competencies and structural sources of role conflict alongside clinical expertise. These findings inform workforce strategies to support performance and sustainability in critical care settings.

## 1. Introduction

Intensive Care Units (ICUs) are among the most demanding clinical environments within modern health-care systems [1]. Respiratory therapists (RTs) working in ICUs are routinely exposed to critically ill patients, high-stakes clinical decisions, emotionally charged situations, and prolonged or irregular work shifts [2]. These conditions require sustained vigilance, rapid judgement, and precise execution of both clinical and non-clinical tasks. Consequently, ICU RTs experience substantial occupational pressure and burnout [3,4], which may compromise their job performance if psychological and organisational demands are not adequately managed.

Job performance in the ICU context is inherently multifaceted [5]. Beyond clinical proficiency, effective performance depends on cognitive focus, emotional regulation, and clear communication under pressure [6]. RTs are required to function as integral members of multidisciplinary teams while managing complex ventilatory care in unstable patients [7]. In such high-risk settings, even modest reductions in performance can adversely affect patient safety, team coordination, and overall quality of care. Identifying factors that support optimal job performance among ICU RTs is, therefore, a critical priority for health-care systems.

Emotional intelligence (EI) has been increasingly recognised as a relevant individual attribute influencing professional functioning in high-stress health-care environments [8]. EI refers to the ability to perceive, understand, and manage emotions in oneself and others [9]. For RTs working in ICUs, where emotional demands are constant and often intense, EI may facilitate emotional regulation, effective interpersonal communication, and sound clinical judgement [10]. The existing literature across health-care disciplines suggests that higher EI is associated with improved professional effectiveness, greater stress tolerance, and adaptive coping behaviours, indicating its potential relevance to job performance across all health-care settings, including critical care units [11,12,13].

In parallel, work–life balance (WLB) represents an important psychosocial factor that may influence performance outcomes [14]. WLB reflects an individual’s capacity to manage work responsibilities alongside personal and social roles [14]. ICU RTs often face challenges in achieving adequate WLB due to shift work, night duties, extended working hours, and disrupted sleep–wake cycles [15]. Poor WLB has been associated with increased fatigue, reduced energy levels, and diminished concentration, all of which may impair clinical efficiency and decision-making. Conversely, better WLB may support sustained attention, motivation, and functional capacity at work, thereby contributing to improved job performance.

Although EI and WLB have each been independently linked to professional outcomes, evidence examining their relationships with job performance among RTs in ICU settings remains limited. Most existing research has focused on physicians or nurses [14,16,17,18], with RTs are frequently underrepresented despite their central role in critical care delivery. Moreover, most available studies originate from non-respiratory contexts, limiting their applicability to the unique demands of ICU RTs. These relationships may also be understood within the Job Demands–Resources (JD-R) model, which suggests that the balance between job demands and available resources shapes job performance. In this context, emotional intelligence may function as a personal resource that supports adaptive functioning under stress, whereas work–life interference represents an additional demand that may adversely affect performance in high-acuity environments [19].

Within the Saudi Arabian health-care system, empirical evidence on these associations is particularly scarce. Cultural expectations, institutional structures, staffing models, and workload patterns may influence how EI and WLB relate to job performance among RTs working in ICUs. The lack of context-specific data limits the development of evidence-based strategies to enhance workforce performance and sustainability in critical care environments. Accordingly, the present study aims to examine the associations between EI, WLB, and job performance among respiratory therapists working in intensive care units in Saudi Arabia. By centring on RTs and their specific clinical context, this study seeks to contribute to a more nuanced understanding of psychological and organisational factors associated with job performance in critical care settings.

## 2. Method

### 2.1. Study Design and Setting

A cross-sectional survey design was employed to examine the associations between EI, WLB and job performance among respiratory therapists working in intensive care units (ICUs). Data were collected between June 2025 and January 2026 from RTs employed in ICUs across multiple hospitals in the Kingdom of Saudi Arabia. The study included governmental and private hospitals to capture variability in ICU practice environments.

### 2.2. Participants and Sampling Procedure

The study population consisted of respiratory therapists currently working in ICUs. A nonprobability purposive sampling approach was used. Eligible participants were RTs actively engaged in ICU clinical practice during the data collection period, with no restrictions based on age, gender, educational level, or years of professional experience.

Participants were recruited using both direct and electronic methods. RTs were approached in person in ICU settings and invited to participate by completing an online questionnaire. In addition, the survey link was disseminated via social media platforms commonly used by RTs in Saudi Arabia, including Twitter (X), WhatsApp, and Telegram, to maximise participation. All responses were collected anonymously using Google Forms (Google LLC, Mountain View, CA, USA; web-based platform with no fixed version number; available at: https://forms.google.com; data collected between [June 2025 and January 2026]. 

### 2.3. Sample Size

A formal sample size estimation was undertaken using the most recent available national workforce data. As of 2024, approximately 5462 respiratory therapy practitioners are registered in Saudi Arabia [20]. Based on this estimate, a minimum sample size of 359 participants was calculated to achieve 95% confidence with a 5% margin of error, assuming a conservative response rate of 50%. In the absence of detailed national data specific to ICU respiratory therapists, the overall workforce estimate was used as a proxy to inform sample size considerations.

### 2.4. Data Collection Instruments

Data were collected using a structured, self-administered questionnaire comprising four sections. The first section collected sociodemographic and professional information, including age, gender, marital status, geographic region, educational level, type of hospital, years of ICU experience, and current shift pattern.

EI was measured using the Wong and Law Emotional Intelligence Scale [21]. This 16-item self-report instrument assesses four domains: self-emotion appraisal, others’ emotion appraisal, use of emotion, and regulation of emotion. Responses are recorded on a 7-point Likert scale ranging from strongly disagree to strongly agree. Subscale scores were computed by summing item responses within each domain, and a total EI score was obtained by summing all items. Higher scores reflect higher EI. The scale has demonstrated strong reliability, with Cronbach’s alpha values ranging from 0.83 to 0.90.

WLB was assessed using the Work–Life Balance Scale developed by Hyman [22]. The scale consists of 15 items covering three dimensions: Work Interference with Personal Life, Personal Life Interference with Work, and Work–Personal Life Enhancement. Responses are rated on a 5-point Likert scale ranging from strongly disagree to strongly agree. For the interference subscales (WIPL and PLIW), higher scores indicate greater interference (i.e., poorer work–life balance), whereas higher scores on the enhancement subscale (WPLE) indicate greater work–personal life enrichment. An overall WLB score was calculated by summing all item scores. The scale has demonstrated good internal consistency, with previously reported Cronbach’s alpha coefficients of 0.91, 0.82, and 0.67 for the respective subscales.

Job performance was assessed using the Individual Work Performance Questionnaire [23]. The IWPQ consists of 18 items measuring task performance, contextual performance, and counterproductive work behaviour. Task and contextual performance items are rated on a 5-point Likert scale from rarely to always, whereas counterproductive behaviour items are rated from never to often. Mean scores were calculated for each subscale. Higher scores on task and contextual performance indicate better job performance, while higher scores on counterproductive work behaviour reflect poorer performance. Reported Cronbach’s alpha coefficients range from 0.78 to 0.85. The instruments used have been widely applied in healthcare research and were administered in English, the standard language of clinical communication among respiratory therapists in Saudi Arabia.

## 3. Data Analysis

Data were analysed using Stata statistical software (StataCorp LLC, College Station, TX, USA), version 17. Descriptive statistics, including means, standard deviations, frequencies, and percentages, were used to summarise participants’ sociodemographic characteristics and the distribution of EI, WLB, and job performance variables. Internal consistency of the study instruments was assessed using Cronbach’s alpha coefficients.

Pearson correlation analyses were conducted to examine bivariate associations between EI, WLB dimensions (work interference with personal life, personal life interference with work, and work–personal life enhancement), and job performance outcomes (task performance, contextual performance, and counterproductive work behaviours).

To examine the independent associations of EI and WLB with job performance, multiple linear regression analyses were performed. Separate regression models were constructed for each job performance outcome. Model 1 included EI as the sole predictor. Model 2 included the three WLB dimensions. Model 3 included EI and all WLB dimensions simultaneously. Unstandardised regression coefficients (B) with 95% confidence intervals were reported. Statistical significance was set at *p* < 0.05. Assumptions of linear regression, including linearity, normality, and homoscedasticity, were assessed and considered acceptable. Multicollinearity was examined using variance inflation factors (VIF), with no evidence of significant multicollinearity.

## 4. Ethical Considerations

Ethical approval was obtained from the Institutional Review Board of King Faisal University, Saudi Arabia (Reference: KFU-REC-2025-FEB-ETHICS3085). Participation was voluntary, and informed consent was obtained electronically prior to survey completion. Participants were informed of the study purpose, procedures, and their right to withdraw at any time without penalty. Anonymity and confidentiality were strictly maintained, with no personally identifiable information collected. All data were securely stored and used exclusively for research purposes.

## 5. Results

### 5.1. Participant Characteristics

A total of 392 respiratory therapists working in intensive care units participated in the study (Table 1). Nearly half were aged 21–29 years (49%), followed by those aged 30–39 years (37.8%); 13.3% were aged ≥40 years. Most participants were male (67.9%). Slightly more than half worked 12 h day shifts (53.6%), and 46.4% worked night shifts. The majority were employed in governmental hospitals (87.5%). Participants were mainly from the Central (36.7%), Western (28.6%), and Eastern (18.4%) regions. Regarding experience, 29.6% had <1 year of ICU experience, while 17.9% had >10 years. Approximately equal proportions were single (49.5%) and married (49.0%).

### 5.2. Descriptive Statistics and Reliability

Descriptive statistics and reliability estimates are presented in Table 2. The mean EI score was 5.36 (SD = 0.98). WLB dimensions showed moderate scores: work interference with personal life (M = 3.09, SD = 0.88), personal life interference with work (M = 2.59, SD = 0.83), and work–personal life enhancement (M = 3.20, SD = 0.75). Mean task performance and contextual performance were 3.71 (SD = 0.87) and 3.49 (SD = 0.98), respectively, while counterproductive work behaviours had a lower mean score (M = 2.17, SD = 0.93). All measures demonstrated acceptable to excellent internal consistency (Cronbach’s α = 0.76–0.93).

### 5.3. Associations Between Emotional Intelligence, Work–Life Balance, and Job Performance

Correlation analyses (Table 3) showed that EI was positively associated with task performance (r = 0.31, *p* < 0.001) and contextual performance (r = 0.36, *p* < 0.001), and negatively associated with counterproductive work behaviours (r = −0.33, *p* < 0.001). Higher EI was also associated with greater work–personal life enhancement and lower work–life interference.

Work–life balance dimensions were significantly related to job performance outcomes. Greater work interference with personal life and personal life interference with work were associated with lower task and contextual performance and higher counterproductive work behaviours. In contrast, work–personal life enhancement showed positive associations with task and contextual performance and negative associations with counterproductive behaviours.

### 5.4. Regression Analyses

Multiple regression analyses (Table 4) showed that EI was positively associated with task performance (B = 0.21, 95% CI 0.09–0.33, *p* < 0.01) and contextual performance (B = 0.30, 95% CI 0.17–0.43, *p* < 0.001), and negatively associated with counterproductive work behaviours (B = −0.24, 95% CI −0.36 to −0.12, *p* < 0.001), after accounting for work–life balance dimensions. Personal life interference with work demonstrated consistent adverse associations, being linked to lower task performance (B = −0.20, 95% CI −0.36 to −0.04, *p* < 0.05) and higher counterproductive work behaviours (B = 0.39, 95% CI 0.23–0.54, *p* < 0.001). However, its association with contextual performance was attenuated and no longer statistically significant in the fully adjusted model (B = −0.13, 95% CI −0.31 to 0.04). In contrast, work–personal life enhancement was positively associated with task performance (B = 0.20, 95% CI 0.04–0.36, *p* < 0.05) and contextual performance (B = 0.20, 95% CI 0.02–0.37, *p* < 0.05). Associations between work interference with personal life and performance outcomes were weaker and largely marginal across models (*p* ≈ 0.10).

## 6. Discussion

The present findings suggest that, in the ICU environment, where rapid decision-making, emotional exposure, and sustained vigilance are routine, professional functioning appears closely tied to both emotional capacity and cross-domain role dynamics. Respiratory therapists who demonstrate stronger emotional awareness and regulation tend to show more stable and constructive performance patterns, whereas personal demands that intrude into the work domain are associated with less favourable behavioural outcomes. At the same time, when professional and personal roles reinforce rather than compete with one another, performance patterns appear more positive. Together, these findings highlight that in high-acuity respiratory care, job performance is not solely a matter of skill execution, but also of emotional management and role balance.

EI appears to play a meaningful role in shaping how respiratory therapists function within the ICU. In this setting, clinical decisions are often made under time pressure, patient instability, and emotional exposure, requiring therapists to sustain both cognitive clarity and professional composure. The findings suggest that the ability to recognise and regulate emotions may support consistent task execution and constructive engagement with colleagues during high-stakes care. Rather than operating as a purely interpersonal skill, EI in the ICU may help stabilise behaviour when clinical demands are intense and continuous. This may reflect the role of emotional regulation in preserving attentional control and reducing cognitive overload under high-acuity conditions. Its inverse association with counterproductive behaviours further indicates that emotional competence is linked to behavioural reliability within critical care teams. These observations are broadly consistent with research among ICU nurses and other health-care professionals [11,24,25], although variability across studies [26] suggests that the strength of this relationship may depend on contextual and role-specific demands.

PLIW emerged as the most consistent adverse factor associated with performance, particularly regarding counterproductive behaviours. In ICU practice, respiratory therapists are required to sustain continuous vigilance and rapid clinical judgement, often in unstable and emotionally demanding situations. When personal responsibilities intrude into the work domain, attentional and emotional resources may become divided, potentially compromising behavioural stability in safety-sensitive environments. This may operate through increased cognitive and emotional load, whereby competing personal demands reduce attentional capacity and consistency in clinical decision-making. Importantly, this association remained significant even after accounting for EI, indicating that individual emotional capacity may not fully offset the impact of cross-domain strain. This pattern is consistent with findings from a meta-analysis, which demonstrate that family-to-work conflict shows stronger, more direct associations with job performance outcomes than work-to-family conflict [27]. Comparable findings have also been reported among clinical nurses [28], reinforcing the relevance of directional work–family dynamics in health-care settings. The association between PLIW and counterproductive work behaviours deserves particular attention, as it produced the largest regression coefficient in the study. In ICU settings, counterproductive behaviours, including reduced communication and diminished task responsiveness, have been linked to increased medical errors and compromised patient safety. Family-to-work conflict has also been shown to impair nurse-assessed patient safety in ICU populations, specifically through cognitive and affective pathways [29]. These findings suggest that cross-domain role interference should be treated not only as a workforce wellbeing concern but as a patient safety priority in critical care respiratory practice.

In contrast, work interference with personal life did not remain independently significant in adjusted models. Although significant at the bivariate level, its predictive value attenuated when EI and other work–life dimensions were considered simultaneously. This directional distinction aligns with evidence from the same meta-analysis indicating stronger performance effects for family-to-work conflict compared with work-to-family conflict [27]. One possible explanation is that long shifts and irregular schedules are structural features of ICU employment that most experienced therapists anticipate and adapt to over time. As such, these demands may carry less acute behavioural impact than unexpected personal intrusions into the workday. However, this interpretation requires prospective examination. These findings underscore the importance of examining direction-specific pathways rather than treating work–life balance as a single, undifferentiated construct.

Work–personal life enhancement showed modest but positive associations with task and contextual performance. When work and personal roles support rather than compete with each other, therapists may experience greater motivation and engagement. This may reflect positive resource spillover, whereby gains in one domain enhance psychological energy and engagement in the other. In ICU settings, being involved in high-stakes, team-based care may strengthen professional identity and a sense of purpose, which can support constructive workplace behaviour. Although these associations were smaller than those observed for interference-based dimensions, positive spillover appears to be linked with more favourable performance patterns. This interpretation aligns with research showing that work–family enrichment is associated with adaptive functioning and engagement [30,31].

Existing research on respiratory therapists in Saudi Arabia has focused largely on burnout and environmental stress, with limited attention to behavioural performance outcomes [4,32]. By examining direction-specific work–life dimensions alongside EI and observable performance domains, the present study adds a behavioural perspective to the national literature. This distinction is particularly important in ICU settings, where performance reliability directly affects patient safety, team coordination, and operational efficiency. Moving beyond wellbeing indicators toward behavioural outcomes provides a more clinically relevant understanding of workforce functioning in high-acuity respiratory practice.

Overall, EI and direction-specific work–life dynamics demonstrated independent yet complementary associations with job performance among ICU respiratory therapists. These associations may also be influenced by unmeasured organisational factors, such as workplace culture, staffing models, and leadership practices. Emotional competence was consistently linked to stable and constructive behavioural patterns, while cross-domain interference—particularly personal life intruding into work—emerged as the most influential adverse factor. Positive spillover showed supportive but more modest associations. Together, these findings suggest that performance reliability in critical care respiratory practice reflects both individual regulatory capacity and the extent to which external demands compete with or reinforce professional functioning.

### Implications for ICU Respiratory Therapy Practice and Workforce Sustainability

Respiratory therapists working in ICUs operate in environments characterised by sustained clinical pressure, high patient acuity, and emotional exposure, all of which increase vulnerability to fatigue and burnout. The present findings suggest that supporting performance in such settings requires attention not only to clinical competence, but also to emotional regulation and cross-domain role strain. Structured opportunities to strengthen communication skills and emotional awareness during professional training may enhance therapists’ capacity to maintain behavioural stability under pressure [33]. While emotional intelligence training has been supported in broader healthcare contexts, evidence specific to respiratory therapy remains limited, and its applicability may not be directly equivalent. However, individual skills alone may be insufficient. Organisational factors—including staffing adequacy, predictable scheduling, and protected recovery time- are likely essential to reduce the spillover of personal demands into clinical functioning [34].

Given the established links between ICU stress, burnout, and workforce attrition in respiratory therapy [3,4,15,32,35,36], addressing both emotional capacity and structural working conditions may contribute not only to improved behavioural performance but also to longer-term workforce sustainability. In high-acuity respiratory care, performance reliability and staff wellbeing are closely intertwined, and strategies aimed at one are unlikely to succeed without consideration of the other.

## 7. Strengths and Limitations

To our knowledge, this study is among the first empirical examinations in Saudi Arabia of the associations between emotional intelligence, direction-specific work–life balance dimensions, and behavioural job performance among respiratory therapists working in intensive care units. No prior study has simultaneously explored these constructs within an ICU respiratory therapy population. By including multiple hospitals across different regions of the Kingdom, the study enhances contextual diversity and strengthens its relevance to national critical care practice. The use of validated instruments and the simultaneous modelling of emotional intelligence and direction-specific work–life balance dimensions allowed assessment of their independent contributions to observable performance outcomes.

Several limitations should be considered. The cross-sectional design limits causal inference, and the findings reflect associations rather than temporal relationships. All measures were self-reported, and while validated instruments were used, some degree of common method influence cannot be entirely excluded. The use of non-probability sampling, including recruitment via social media platforms, may have influenced participation patterns; however, efforts to recruit across multiple regions and institutions were intended to enhance sample diversity.

The sample was relatively young, consistent with national workforce data indicating that the majority of respiratory therapists in Saudi Arabia are under 40. The sample was predominantly male, which contrasts with national data, which suggests a more balanced gender distribution. This difference may reflect participation patterns or recruitment dynamics; however, it does not affect the internal consistency of the observed associations between emotional intelligence, work–life balance, and job performance. In addition, the study focused on respiratory therapists working in ICU settings, and the findings may not extend to other clinical contexts.

The analytical approach did not include adjustment for additional covariates, as the study aimed to examine primary associations between key constructs while preserving model interpretability. Unmeasured organisational factors, such as workplace structure and staffing context, may also contribute to performance variability. Interaction effects between emotional intelligence and work–life balance were not examined and may represent an area for future research. Cultural and organisational context within the Saudi healthcare system should also be considered when interpreting the findings.

## 8. Conclusions

Among respiratory therapists working in intensive care units, job performance appears to depend on more than clinical skill alone. In high-acuity environments characterised by patient instability, rapid decision-making, and sustained emotional exposure, professional functioning is closely linked to both emotional capacity and cross-domain role dynamics. EI was consistently associated with more stable and constructive performance patterns, while personal life interference with work emerged as the most influential adverse factor, highlighting the impact of competing external demands on behavioural reliability. These findings indicate that sustaining consistent and effective performance in ICU settings requires attention not only to clinical competence, but also to emotional regulation and the management of role strain beyond the workplace. By focusing on an underrepresented professional group, this study extends workforce research toward a more comprehensive understanding of performance in critical care environments.

## Figures and Tables

**Table 1:** Demographics of the study respondents (n = 392).

Variable	
Age, n (%)	
21–29	192 (49%)
30–39	148 (37.8%)
40 and above	52 (13.27%)
Gender (male %)	266 (67.9%)
Shift schedule, n (%)	
12 h Day shift	210 (53.6%)
12 h Night shift	182 (46.4%)
Type of hospital n (%)	
Governmental	343 (87.5%)
Private	49 (12.5%)
Geographical Region, n (%)	
Eastern	72 (18.4%)
Central	144 (36.7%)
Western	112 (28.6%)
Southern	48 (12.24%)
Northern	16 (4.1%)
Years of clinical experience, n (%)	
<1 year	116 (29.6%)
1–5 years	112 (28.6%)
6–10 years	94 (24%)
>10 years	70 (17.9%)
Marital status, n (%)	
Single	194 (49.5%)
Married	192 (49%)
Divorced/separated/widowed	6 (1.5%)
Data are presented as n (%), unless stated otherwise.	

**Table 2:** Descriptive Statistics and Reliability of Main Study Variables (n = 392).

Variable	Mean	SD	Cronbach’s α
Emotional Intelligence (Total)	5.36	0.98	0.93
Work Interference with Personal Life (WIPL)	3.09	0.88	0.91
Personal Life Interference with Work (PLIW)	2.59	0.83	0.85
Work–Personal Life Enhancement (WPLE)	3.20	0.75	0.79
Task Performance	3.71	0.87	0.82
Contextual Performance	3.49	0.98	0.92
Counterproductive Work Behaviours	2.17	0.93	0.76

Higher scores indicate higher emotional intelligence and job performance. For work–life balance dimensions, higher scores on WIPL and PLIW indicate greater interference, whereas higher scores on WPLE indicate greater work–personal life enhancement. Higher scores on counterproductive work behaviours indicate more frequent negative behaviours. All scales demonstrated acceptable to excellent internal consistency.

**Table 3:** Correlations Between Emotional Intelligence, Work–Life Balance, and Job Performance (n = 392).

Variable	1	2	3	4	5	6	7
1. Emotional Intelligence (EI)	—						
2. Work Interference with Personal Life (WIPL)	−0.11 *	—					
3. Personal Life Interference with Work (PLIW)	−0.13 *	0.46 ***	—				
4. Work–Personal Life Enhancement (WPLE)	0.20 ***	−0.19 ***	0.12 *	—			
5. Task Performance	0.31 ***	−0.28 ***	−0.27 ***	0.22 ***	—		
6. Contextual Performance	0.36 ***	−0.23 ***	−0.19 ***	0.22 ***	0.73 ***	—	
7. Counterproductive Work Behaviours	−0.33 ***	0.33 ***	0.42 ***	−0.15 **	−0.17 ***	−0.10 *	—

Values represent Pearson correlation coefficients. *p* < 0.05 *, *p* < 0.01 **, *p* < 0.001 ***. Higher scores indicate higher levels of emotional intelligence, work–life balance, and job performance. Higher scores on counterproductive work behaviours indicate more frequent negative behaviours.

**Table 4:** Regression Models Examining the Associations of Emotional Intelligence and Work–Life Balance with Job Performance.

Predictor	Model 1: B (95% CI)	Model 2: B (95% CI)	Model 3: B (95% CI)
Panel A. Task Performance			
Emotional Intelligence (EI)	0.28 (0.16, 0.40) ***	—	0.21 (0.09, 0.33) **
Work Interference with Personal Life (WIPL)	—	−0.14 (−0.29, 0.02) ^†^	−0.14 (−0.29, 0.01) ^†^
Personal Life Interference with Work (PLIW)	—	−0.24 (−0.40, −0.08) **	−0.20 (−0.36, −0.04) *
Work–Personal Life Enhancement (WPLE)	—	0.26 (0.10, 0.42) **	0.20 (0.04, 0.36) *
R^2^	0.096	0.149	0.202
Adjusted R^2^	0.094	0.143	0.193
Panel B. Contextual Performance			
Emotional Intelligence (EI)	0.36 (0.22, 0.49) ***	—	0.30 (0.17, 0.43) ***
Work Interference with Personal Life (WIPL)	—	−0.13 (−0.30, 0.05)	−0.13 (−0.30, 0.04)
Personal Life Interference with Work (PLIW)	—	−0.19 (−0.37, −0.01) *	−0.13 (−0.31, 0.04)
Work–Personal Life Enhancement (WPLE)	—	0.28 (0.10, 0.46) **	0.20 (0.02, 0.37) *
R^2^	0.128	0.105	0.190
Adjusted R^2^	0.126	0.098	0.181
Panel C. Counterproductive Work Behaviours (CWB)			
Emotional Intelligence (EI)	−0.31 (−0.44, −0.19) ***	—	−0.24 (−0.36, −0.12) ***
Work Interference with Personal Life (WIPL)	—	0.13 (−0.03, 0.29)	0.13 (−0.02, 0.28) ^†^
Personal Life Interference with Work (PLIW)	—	0.43 (0.27, 0.59) ***	0.39 (0.23, 0.54) ***
Work–Personal Life Enhancement (WPLE)	—	−0.21 (−0.38, −0.05) *	−0.15 (−0.31, 0.01) ^†^
R^2^	0.109	0.226	0.284
Adjusted R^2^	0.107	0.220	0.276

Values represent unstandardised regression coefficients (B) with 95% confidence intervals (CI). Model 1 includes only emotional intelligence (EI). Model 2 includes only work–life balance dimensions: work interference with personal life (WIPL), personal life interference with work (PLIW), and work–personal life enhancement (WPLE). Model 3 includes EI and all work–life balance dimensions simultaneously. Higher scores indicate higher levels of emotional intelligence, work–life balance, and job performance. Higher scores on counterproductive work behaviours indicate more frequent negative behaviours. R^2^ indicates the proportion of variance in the outcome explained by each model. ^†^ *p* < 0.10, *p* < 0.05 *, *p* < 0.01 **, *p* < 0.001 ***.

## Data Availability

The data presented in this study are available on request from the corresponding author. The data are not publicly available due to privacy and ethical restrictions.
